# Recent Advances in Real-Time Label-Free Detection of Small Molecules

**DOI:** 10.3390/bios14020080

**Published:** 2024-02-01

**Authors:** Andy Chieng, Zijian Wan, Shaopeng Wang

**Affiliations:** 1 Center for Bioelectronics and Biosensors, Biodesign Institute, Arizona State University, Tempe, AZ 85287, USA; achieng1@asu.edu (A.C.); zwan13@asu.edu (Z.W.); 2 School of Molecular Science, Arizona State University, Tempe, AZ 85287, USA; 3 School of Biological and Health Systems Engineering, Arizona State University, Tempe, AZ 85287, USA

**Keywords:** small molecule, label-free, real-time detection, biosensor, optical transduction, electrochemical transduction, piezoelectric transduction

## Abstract

The detection and analysis of small molecules, typically defined as molecules under 1000 Da, is of growing interest ranging from the development of small-molecule drugs and inhibitors to the sensing of toxins and biomarkers. However, due to challenges such as their small size and low mass, many biosensing technologies struggle to have the sensitivity and selectivity for the detection of small molecules. Notably, their small size limits the usage of labeled techniques that can change the properties of small-molecule analytes. Furthermore, the capability of real-time detection is highly desired for small-molecule biosensors’ application in diagnostics or screening. This review highlights recent advances in label-free real-time biosensing technologies utilizing different types of transducers to meet the growing demand for small-molecule detection.

## 1. Introduction

Small molecules, typically defined as molecules under 1000 Da, have been of great interest due to their ubiquitous presence in biological processes and their intrinsic properties in human anatomy [1,2]. Notably, small molecules are capable of crossing the blood-brain barrier and have relative ease in cell membrane permeation compared to larger molecules; thus, small molecules can both enter the body readily and traverse to targets with high specificity [1,2]. These properties have great appeal in the pharmaceutical industry, with 15 of the 37 drugs approved by the FDA in 2022 being small molecules [3]. On the other hand, the properties that make small molecules appealing for drug development also make them effective toxins, carcinogens, mutagens, and endocrine disruptors both natural, such as several secondary metabolites of fungi, cyanobacteria, plants, and other organisms; and artificial, such as chemical warfare agents [1,2,4,5,6,7]. Furthermore, biological systems themselves utilize small molecules such as amino acids, steroids, sugars, or metabolites for many of the processes essential for life [1,2,7].

**Figure 1:**
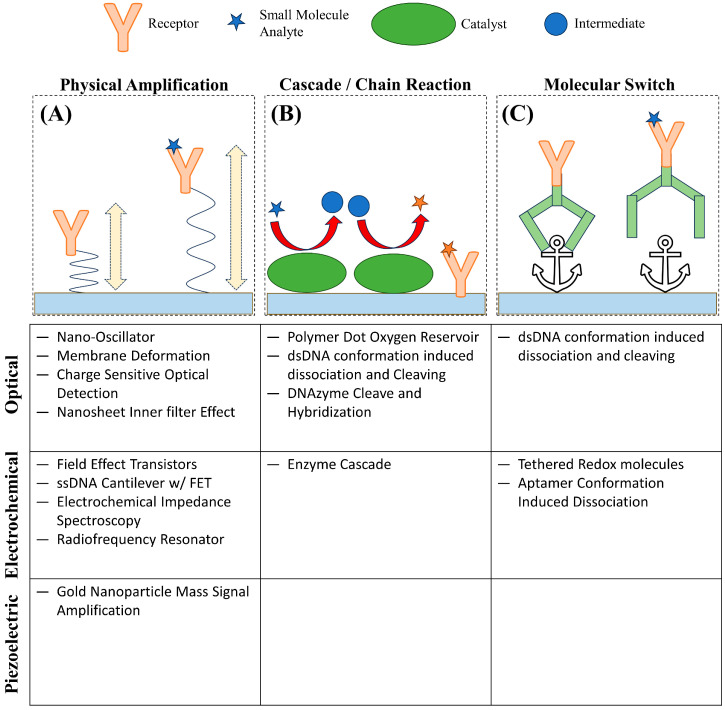
Representative novel strategies for small molecule detection. (**A**) Small-molecule binding induces a change in amplitude of a charge-sensitive oscillation. (**B**) A small molecule analyte undergoes a series of reactions to produce a product that can be detected. (**C**) Small-molecule binding results in a conformational change releasing a structure from an anchor point.

Considering the biological importance of small molecules, the development of biosensors capable of real-time detection has been in great demand with widespread applications such as: ensuring correct dosage of drugs, early diagnosis of disease, or continuous detection of toxins. Biosensors are generally composed of a receptor that captures an analyte and a transducer that changes in property upon the binding between the analyte and the receptor to produce a recordable signal [2]. Unfortunately, due to the low abundance and mass of small molecules, many techniques, especially those where the transducer’s signal strength scales with analyte mass, are challenging to achieve the sensitivity necessary for the effective detection of small molecules under biologically relevant conditions [7,8]. In addition, label-free techniques are greatly preferred for small molecules, even more so compared to larger analytes like proteins. The low mass and small size of small molecules only exacerbate the concerns of labeled detection techniques in that labels such as fluorescent proteins, fluorescent dyes, and quantum dots can alter the binding behavior and physical properties of an analyte [9,10]. In many cases, the label is much larger than the analyte itself; for example, green fluorescent protein, a common fluorescent label, has a molecular weight of 27 kDa to 69 kDa, and even small-molecule organic fluorescent probes such as fluorescein have a molecular weight of approximately 332 Da; a very significant addition to a small molecule with mass less than 1000 Da [9,10,11,12]. Currently, one of the common techniques for molecular detection is enzyme-linked immunosorbent assay (ELISA) and its variations, which offers high accuracy and sensitivity and can be used for small molecule detection; however, ELISA is an endpoint detection method that does not provide real-time kinetic information [13]. Small-molecule interaction kinetics and affinity with their target or receptor molecules are essential information for studying small molecule functions [14]. Thus, real-time measurement is preferred; correspondingly, transduction methods that can be measured continuously, such as changes in refractive index, surface conductance, and resonance frequency, have been developed into widespread label-free techniques, including surface plasmon resonance (SPR), field effect transistor (FET), and quartz crystal microbalance (QCM), for real-time small molecule detection using the corresponding receptor or target molecules as recognition elements [2,15,16,17,18,19,20]. The unique challenges to achieving the sensitivity and selectivity necessary to detect small molecules are leading to innovations to amplify signal and optimize surface chemistry as well as interesting methods to indirectly measure binding. These innovations can be broadly categorized as: physical amplification, cascading or chain reaction, and molecular switches (Figure 1). These strategies attempt to circumvent the limiting attributes of small molecules by measuring the change of alternative properties upon binding, correlating the concentration of analyte to a more easily measured product, or using a conformational change induced by analyte binding to produce a signal, respectively.

In this review, we explore recent advances in label-free real-time biosensing techniques utilizing optical, electrochemical, and piezoelectric transducers and the innovations or adaptations to meet the growing demands of small-molecule detection. Specifically, we will cover works that provide innovative solutions to the challenges of detecting small molecules that can reasonably be applied in biosensors utilized in point-of-care diagnostics and real-time screening. Ultimately, the goal of developing small-molecule detection is ensuring the resulting technology can serve a need. Although it is important for the sensitivity and selectivity of instrumentation to be advanced, other considerations such as practicality, affordability, and accessibility are factors that must be considered for the development of miniaturized, cost-effective biosensors for point-of-care application and are equally important. Although reviews of label-free biosensing technologies are readily available [21,22,23], we offer a perspective on challenges and advancements that specifically target the detection of small molecules under 1 kDa. Recent reviews on small-molecule detection have been focused on advancements of particular transducers or particular small molecules of interest [2,4,5,6,7,16]. Therefore, this review offers a comprehensive introduction to the recent advancement in label-free and real-time small-molecule detections since the last comprehensive review to our knowledge [1].

## 2. Optical Transduction

Optical transducers have great potential in the real-time detection of small molecules, but also face intrinsic challenges to fully utilizing their advantages. Optical transductions can measure a variety of signals related to the presence of small molecules, such as absorption, scattering, luminescence, or refractive index [2]. Optical biosensors typically offer fast results with high temporal resolution for real-time monitoring of binding, making them ideal for diagnostics and point-of-care devices [7]. However, the signal of optical transducers generally scales with the size and/or mass of the analyte of interest, especially when avoiding the use of fluorescent labels [2,7,24]. For example, one of the commonly used label-free optical detection techniques, surface plasmon resonance, has been lauded as the gold standard for molecular binding kinetic measurement, being used for analytes such as proteins, DNA, RNA, peptides, and other biological macromolecules, but attempts to utilize SPR for the detection of small molecules required advanced instrumentation or significant enhancement of receptor surface [15,25]. Thus, the development of optical techniques for the measurement of small molecules has primarily focused on either enhancing the weak signal of small-molecule binding or by measuring binding indirectly via measuring phenomena induced by analyte-binding events.

Surface plasmon resonance utilizes surface plasmons, electron oscillations at a metal-dielectric interface, typically gold-coated glass, which respond via oscillation at resonance with a light wave [26]. The evanescent waves of this oscillation are sensitive to changes close to the metal surface, notably as a change in refractive index due to the binding of an analyte, which shifts the SPR signal and produces a signal proportional to the analyte’s mass [27]. Unfortunately, this signal dependency on the mass of an analyte makes it challenging for SPR to detect small molecules, often requiring enhancement of either binding site density or signal strength such as through the usage of dextran chips or by utilizing localized surface plasmon resonance with nanostructures [15]. Thus, there has been great interest in developing sensing platforms with high sensitivity to small molecules, requiring simpler sample preparation and instrument operation, and for diagnostic use, having high portability and low cost.

**Figure 2:**
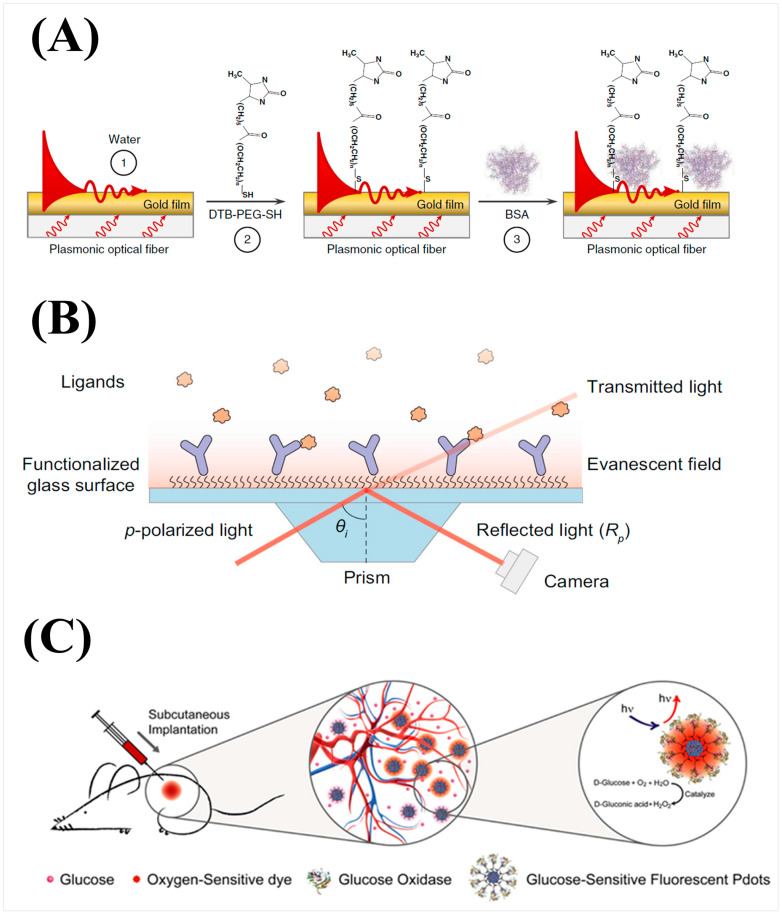
(**A**) Fabrication of gold-coated optical fiber to produce superfine plasmonic spectral combs [31]. (**B**) Schematic of critical angle reflection microscopy [34]. (**C**) Schematic of implantable polymer-dot transducer adapted with permission from [35]. Copyright 2016 American Chemical Society.

Since the emergence of SPR in the 1970s, surface plasmon-based techniques have been developed with great interest, and different strategies have emerged to expand techniques into the range of small molecules and overcome their inherent mass dependency [28]. The use of gold-coated optical fibers instead of traditional gold chips for SPR has been in development since the 1990s, offering reduced cost and size compared to traditional SPR; which facilitates its application in point-of-care or diagnostic fields and the ability to optimize sensor attributes to suit the sensor’s purpose by changing the fabrication of the fiber probe [29,30]. For example, Liu et al. developed a sensor capable of detecting estradiol, a small-molecule drug and hormone, by utilizing fiber grafting to produce high-sensitivity narrow cladding mode spectral combs, shown in Figure 2A, which increased the resolution of the refractive index from 10^−6^ to 10^−8^ RIU (refractive index unit) and displayed minimal temperature sensitivity by calibrating the light within the fiber core, and reached a limit of detection (LOD) of 1.5 × 10^−12^ g/mL [31]. A different approach to the utilization of surface plasmons for detection is exploiting the rapid decay of evanescent waves as distance increases from the sensor surface. Nano-oscillators have been developed in which a receptor is tethered to the surface with a polymer chain and oscillated using an alternating electric field [32,33]. Upon binding to the receptor, the charge of the receptor and thus the oscillation amplitude is changed resulting in a change in the oscillation amplitude and the scattering of plasmonic waves that is reflected in the intensity of the plasmonic imaging [32,33]. This surface plasmon technique thus circumvents the mass dependency of traditional SPR by instead relying on the charge dependency of the oscillation amplitude. HSV-1 virions tethered to polyethylene glycol linkers and nanodisc encapsulated membrane protein KcsA-Kv1.3 tethered to DNA linkers were able to detect tocrifluor at a detection limit of 16 molecules per virion and 4-(2-ethylpiperidin-1-yl)-2-methyl-6-phenyl-5H-pyrrolo [3,2-d]pyrimidine (EMPPP) at a detection limit of 4.0 × 10^−15^ g/mm^2^, respectively [32,33].

Although SPR is an effective surface sensitive tool with growing capability for small molecule detection, other evanescent optical sensing principles have been utilized as alternatives. One class of them is grating-based optical biosensors, including commercial products, such as Corning Epic or Malvern Creoptix. In general, they have similar performance and cost compared to SPR based systems [36,37,38]. An example of recent development in this class of sensor is guided-mode resonance (GMR), which is also a kind of grating structure-based refractive index sensor [39]. This reported system uses an LED light source and photodetector for reduced cost and improved portability. The small changes of guided-mode resonance wavelength shifts are converted into changes in light intensity, allowing real-time quantification of dinitro-phenyl (DNP) with a LOD of 7.5 × 10^−8^ g/mL [39]. Both SPR and grating-based systems need special nanofabricated sensor chips that represent considerable cost for consumables. Critical Angle Reflection (CAR) is another recently developed alternative method that measures molecular interaction-induced refractive index change via change in reflectivity when the incident light is close to the critical angle, the angle at which the incident light produces an angle of refraction of 90° [34]. CAR instrumentation is virtually the same as conventional SPR as shown in Figure 2B and utilizes bare cover glass rather than gold chips, allowing easy implementation where conventional SPR is practiced and at reduced cost [34]. At high incident angles, CAR surpasses SPR in sensitivity by up to five times, but at the cost of reducing dynamic range, and was able to measure small drugs such as furosemide, sulpride, and methyl sulfonamide with a LOD as low as 1.5 × 10^−12^ g/mm^2^ [34].

Evanescent techniques have been a great asset in molecular detection with their high sensitivity, but these techniques typically struggle with the detection of small molecules due to their signal strength being mass dependent. Thus, some recent works have been exploring biosensing platforms that detect phenomena induced by small-molecule binding to indirectly measure its binding kinetics. One approach is to measure molecular binding-induced nanoscale cell membrane deformation on a whole cell assay. Single cells are immobilized on a substrate for the detection of small molecules binding to cell outer membrane receptors, e.g., acetylcholine binding to nicotinic acetylcholine receptors [40]. Upon binding of the small molecules, the cell membrane is deformed proportionally to the number of ligands bound to receptors. The nanometer scale deformation is detected precisely via a simple differential detection algorithm that calculates the change in relative image intensity within and outside of the cell edge region [40]. Note that cells can be live or fixed, as the deformation is caused directly by the binding events, not downstream events, suggesting this technique may lead to platforms both for the detection of small molecules and for tracking interactions between cellular membranes and proteins of interest. Another approach utilized an optical fiber suspended over a camera or position-sensitive diode, and is oscillated by an applied alternating current with amplitude proportional to the surface charge of the optical fiber [41,42,43]. Upon binding of analyte onto the surface, the surface charge density of the optical fiber is changed, producing a quantifiable signal that is dependent on the charge of the molecule rather than the mass of the molecule. The method has a LOD of 0.14 e^−^/μm^2^ for imatinib, a small-molecule drug. This charge-sensitive optical detection method is an appealing alternative for label-free detection of small-molecule binding kinetics [41,42,43].

Although its classification of being label-free is debated, it is worth considering fluorescence-based techniques in which the analyte is not directly labeled. Fluorescence techniques have been highly popular due to their high sensitivity and rapid data analysis. Though many fluorescence techniques rely on labeling analytes with dye molecules, fluorescent techniques that exclude the labeling process can utilize excited auto-fluorescence or analyte-induced quenching to avoid directly modifying analyte behavior [1,44]. However, these techniques struggle with analytes having high electron affinity when using photo-induced electron transfer for excitation [45]. A graphitic carbon nitride semiconductor nanosheet layer was used to overcome this issue for the detection of picric acid, a nitroaromatic explosive that can accumulate in water, and shows great promise in utilizing the high sensitivity that fluorescent techniques offer. By exploiting the strong inner filter effect, which is typically considered a hinderance that reduces fluorescence signal, between picric acid and the nanosheets to quench nanosheet fluorescence upon binding of picric acid, an LOD of approximately 1.9 × 10^−9^ g/mL was obtained [45]. Another approach is the use of binding-induced cleavage to measure small-molecule binding events. A CRISPR-Cas12a derived biosensing platform was developed in which upon small-molecule binding onto aTFs proteins bound to double-stranded DNA (dsDNA), the dsDNA is dissociated due to the resulting conformation change of aTF. The free dsDNA can then bind to a Cas12a-crRNA complex resulting in activation of Cas12a. The cleaving of a fluorescence quencher labeled single-stranded DNA (ssDNA) by the activated Cas12a induced a change in fluorescent intensity proportional to the small-molecule concentration and binding process [46]. Although this technique uses a molecular label, the label is applied to an indirectly related ssDNA. This approach allows the use of highly sensitive fluorescence techniques with minimal impact on the binding and properties of the analyte, obtaining a LOD of 1.68 × 10^−9^ g/mL for uric acid detection [46]. Another example of indirect fluorescence signal is the use of fluorescent semiconductor polymer dots that were developed as nanoparticle sensing platforms [35]. A polymer dot transducer was developed in which an enzyme-catalyzed reaction with glucose would deplete an internal oxygen reservoir resulting in a fluorescent signal visible through skin, allowing in vivo detection of glucose in cells and tissue with an LOD of ~2–8 × 10^−3^ g/mL as portrayed in Figure 2C [35]. This technology can apply to many oxygens consuming enzyme-related small molecules as a means for in vivo detection in patients as an alternative to electrochemical methods that have similar speeds, size, and convenience, but require extracted bodily fluid samples for each measurement [47].

One unique approach in the development of an optical biosensor for small molecules is the liquid crystal biosensor, which has emerged for small-molecule detection recently [48]. Liquid crystals have a long-range orientational order that is sensitive to change at the binding interface allowing label-free high-sensitivity detection. However, the optical signal of liquid crystal sensors is generally proportional to the size and number of biomolecules producing topographical changes on glass slides, and thus were not used for small molecules [48]. By modifying a glass slide using DNAzyme, upon cleaving by L-histidine, a partial substrate sequence is realized and hybridizes with the capture probe producing a DNA duplex on the surface, thus inducing an amplified optical signal compared to the small molecule directly binding to a capture probe with an LOD of 7.8 × 10^−3^ g/mL and displaying the potential value in further exploring liquid crystal biosensors for small-molecule detection [48].

## 3. Electrochemical Transduction

Electrochemical transducers share many of the advantages of optical transducers in that they also offer fast, real-time measurements of binding and have the added benefit of generally requiring low-cost instrumentations that can be designed for high portability and accessibility for use with little to no training. Furthermore, the signal of electrochemical transducers is generally not significantly dependent on the size or mass of analytes, but rather on their electronic properties, such as electrochemical reactivity or charge [16,49,50]. Traditionally, electrochemical biosensors utilized potentiometry or amperometry to detect redox reactions at an electrode surface, offering a mass-independent signal, but this greatly limits the range of molecules that can be detected via electrochemical biosensors to electroactive molecules only. Thus, although real-time electrochemical detection of oxygen and glucose has already reached the commercialization stage for diagnostics and point-of-care monitoring, the detection of other small molecules through electrochemical detection is still developing [47]. For this reason, innovations in the field have explored other means for analytes to modify the electrical properties of transducers. In particular, impedance-based transducers are promising alternatives that are primarily dependent on the charge of small molecules. Thus, impedance biosensors such as FET sensors have grown in popularity for the detection of small molecules due to their detection mechanism, in which the signal is produced by a change in conductance upon binding of charged molecules, allowing high-sensitivity mass independent measurement [17,18]. Unfortunately, charge-sensitive techniques face challenges from nonspecific binding and Debye screening from the biologically relevant high ionic strength that hinder their sensitivity in serum, plasma, or even phosphate-buffered saline (PBS) [6,51]. Sensing platforms for small molecules have developed methods to use electrochemical transduction that can detect a larger range of different analytes by enhancing the signal strength of impedance-based biosensors to compensate for charge screening in biologically relevant ionic concentrations. Furthermore, enhancement of signal strength increases the sensitivity of electrochemical instrumentation, and helps to detect the relatively low abundance small molecules typically have in nature [52,53].

**Figure 3:**
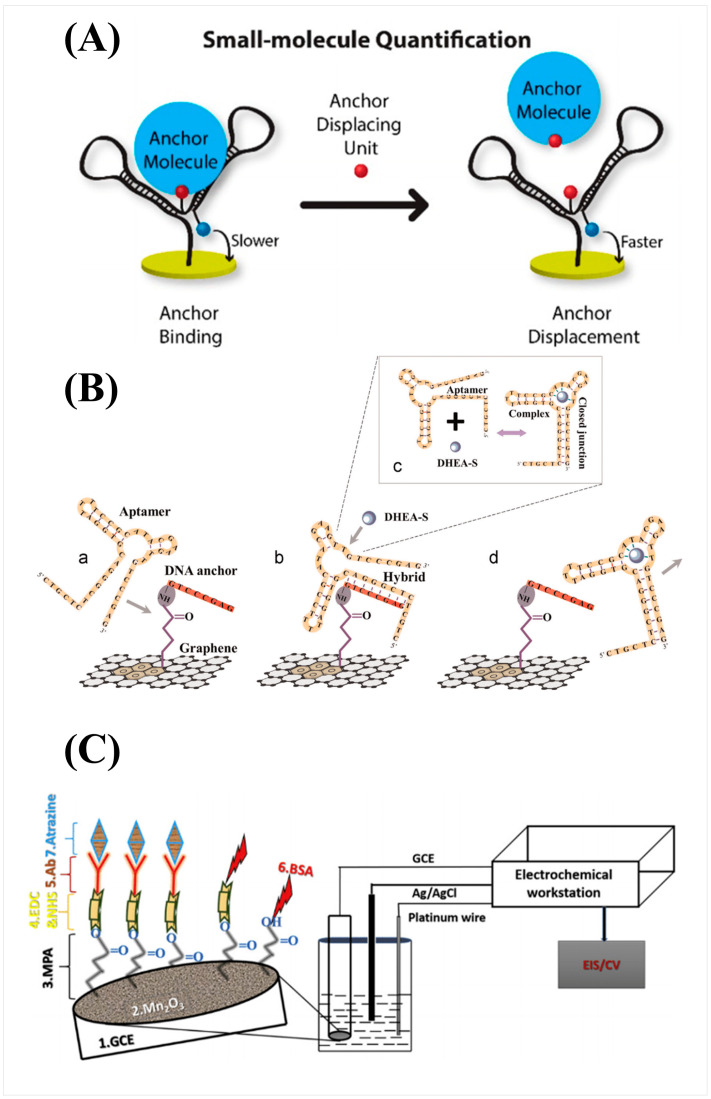
(**A**) Schematic of small-molecule DNA nanostructure in which anchor displacement results in faster diffusion of tethered redox molecule and increased current adapted with permission from [56]. Copyright 2019 American Chemical Society. (**B**) Schematic of graphene nanosensor in which binding changes conformation of aptamer disrupting hybridization and releasing aptamer from surface reprinted from [61], Copyright (2015), with permission from Elsevier. (**C**) Schematic of proposed biosensor using electrospun Mn_2_O_3_ nanofibers. Reprinted from [64], Copyright (2019), with permission from Elsevier.

One of the weaknesses of electrochemical detection, particularly in amperometry or potentiometry, is that the analyte of interest must be electrochemically active in order to produce a signal. One workaround to this weakness adapted commercially personal glucose meter for the detection of ATP by using a cascade enzymatic reaction promoted by hexokinase and pyruvate kinase [54]. The amount of ATP is inversely proportional to glucose through the catalyzation of glucose to glucose 6-phosphate in which ATP is converted to ADP [54]. Pyruvate kinase catalyzes the regeneration of ATP from ADP to further react glucose and amplify the signal. Concentrations of ATP as low as 2.5 × 10^−8^ g/mL can be detected [54]. This technique offers a potential means to indirectly measure small molecules that are not electroactive by instead measuring the proportional signal of an electroactive product from an enzymatic reaction. Kurbanoglu et al. developed a methimazole (MT) enzyme cascade blocking biosensor using a nanocomposite of magnetic nanoparticles and iridium oxide nanoparticles on screen-printed electrodes and obtained an LOD of 6.85 × 10^−10^ g/mL [55]. By utilizing the inhibition of tyrosinase via chelating copper and forming thioquinone with MT, the concentration of MT can be measured via amperometry resulting in a miniaturized lab on a chip biosensor that can be adapted to other small molecules that can inhibit enzymes [55]. One innovative approach utilized a customized DNA nanostructure attached at a fixed distance from an electrochemical transducer surface with an attached redox-active molecule as shown in Figure 3A [56]. Upon analyte binding, the change in mass shifts the tethered diffusion between the redox-active molecule and the electrochemically active surface, offering an interesting means to use well-studied redox reaction measurements for small molecule detection. An increased current results from the faster diffusion and reduced distance between the redox-active molecule and the transducer surface. An LOD of 9.0 × 10^−7^ g/mL and 6.9 × 10^−5^ g/mL for biotin and digoxigenin were obtained, respectively [56]. DNA nanostructures are particularly compatible with aptamers, functionalized single-strand nucleic acids that can have high selectivity to analytes of interest through Systematic Evolution of Ligands by Exponential Enrichment (SELEX) for rapid selection of random sequences [57,58]. Notably, an aptamer with a DNA triplex assembled on nanotetrahedron on screen-printed electrodes was utilized for the detection of saxitoxin and obtained an LOD of 2.8 × 10^−10^ g/mL [59]. Upon small-molecule binding, the switch of aptamer triplex lead to dissociation of the pyrimidine arms from the electrode and induced an increase in current that measured with square wave voltammetry and CV [59].

Field Effect Transistors have been popular candidates for small-molecule detection because they are charge-sensitive rather than mass-sensitive. However, they face challenges in biologically relevant conditions due to reduced sensitivity in high ionic concentration, which shields the charge of analyte molecules [17]. Strategies have been developed to enhance the detection of small molecules, particularly those with weak surface charge. A single walled carbon nanotube-based FET biosensor was developed as a sensitive, portable method to detect norfentanyl, a primary metabolite of fentanyl [60]. Although the detection of norfentanyl can offer more reliable results than detecting fentanyl due to having a longer detection window, norfentanyl is not electroactive, limiting the electrochemical techniques capable of its detection [60]. By decorating the single-walled carbon nanotube surface with gold nanoparticles, nonspecific binding is reduced, but at the cost of reduced sensitivity compared to direct coupling of antibody to carbon nanotube surface [60]. A reduced norfentanyl antibody was also found to enhance sensitivity by controlling orientation and bringing the binding closer to the surface [60]. An aptameric graphene field-effect transistor sensor was developed by Wang et al. for the detection of small molecules [61]. Analyte-specific aptamers were hybridized onto the sensor surface. Analyte binding releases the aptamer anchor and induces a large charge change due to the highly charged nature of aptamers as shown in Figure 3B. A strong signal can be obtained in this way with an LOD of 1.65 × 10^−8^ g/mL [61]. Another approach sought to overcome the charge screening limitation of FET by utilizing oligonucleotide receptors with aptamers screened with adaptive loop binding on nanometer-thin In_2_O_3_ to detect dopamine, serotonin, glucose, and sphingosine-1-phosphate [62]. Nucleic acid-based receptors have proven to supplement FET surface chemistry as an alternative to traditional antibodies due to their high selectivity and control over their structure and orientation. To exemplify the versatility of nucleic acid structures, an electromechanical detector using aptamer probes bound to a ssDNA cantilever attached to a double-stranded DNA tetrahedral structure [63]. An alternating electric field induces the cantilever to raise and lower, which can be monitored after attachment of a fluorescent dye cyanine3 at the tip of the cantilever and further measured in parallel using a graphene layer and a field-effect transistor to detect ATP [63]. Although a fluorescent dye is utilized, it labels the cantilever rather than the analyte of interest thus providing the benefits and ease of measuring fluorescence without the downsides of labeling analyte.

Another impedimetric technique, electrochemical impedance spectroscopy (EIS), measures the charge transfer resistance of an electrode surface upon binding. EIS has recently gained the capability to detect small molecules using modified sensing layers with enhanced electronic properties with high selectivity and sensitivity for change in impedance such as with aptamers or molecularly imprinted polymers (MIP) [51]. Hui Lee et al. developed nanoscale molecularly imprinted composite polymer with a thickness of less than 5 nm as an alternative to traditional thicker receptors, offering synthesized thinner layers with higher target to receptor ratios, and resulting in increased sensitivity with an LOD of 5.76 × 10^−10^ g/mL for cortisol [65]. A quantum electrochemical impedance spectroscopy technique was used to measure resonant quantum conductance changed induced by cortisol binding, because the change in charge-transfer resistance was insufficient for traditional electrochemical impedance spectroscopy to detect, due to the low resistance of the thin MIP film [65]. Another approach, metal oxide nanofibers have grown in popularity as biosensing material due to their high sensitivity and electronic properties. A Mn_2_O_3_ nanofiber was developed by Supraja et al. as a low bandgap working electrode for the EIS detection of atrazine with full setup in Figure 3C and a proposed LOD of 2.2 × 10^−22^ g/mL that can be used to develop real-time detection biosensors of water samples [64]. A high throughput impedimetric sensing platform was developed by using peptide aptamers screened via molecular docking in silica to determine the potential interaction between L-arginine and the extracted peptide aptamer for specificity and binding energy and obtained an LOD of 1.92 × 10^−16^ g/mL [66]. This represents a potential model for future electrochemical impedance spectroscopy-based sensor design workflow as different molecular docking groups can be quickly screened in silica before validation by a well-established experimental method such as isothermal titration calorimetry and then be easily incorporated into an assay for small molecules [66].

One of the most common targets for small-molecule electrochemical detectors is glucose, an important molecule both as a source of energy and a biomarker of diabetes. The detection of glucose has advanced greatly over the years due to the high demand of patients that regularly need to check blood sugar levels [47]. Thus, miniaturization and accessibility are particularly high priorities for glucose detection, both features that electrochemical transducers excel at offering. Radiofrequency-based biosensors have been an interesting alternative impedimetric technique that measures glucose concentrations by measuring the level of electromagnetic coupling dependent on glucose permittivity [67]. A miniaturized RF resonator biosensor was recently developed using an interdigital capacitor embedded between two divisions of a spiral-inductor allowing high sensitivity of glucose detection both in water and in serum with LOD of 1.1 × 10^−8^ g/mL and 5.9 × 10^−9^ g/mL, respectively, without the need for mediators commonly used in enzymatic glucose sensors that can degrade performance [67].

As previously mentioned, small molecules have low mass and size, but another common feature of small molecules is their relatively low abundance in their native environments [52,53,68]. Most studies attempted to overcome this challenge by improving surface chemistry for better capture efficiency and selectivity. However, Cui et al. developed an interesting approach to improve sensitivity to low concentrations of small molecules by increasing the transport rate of the analyte to the sensor surface [69]. Most biosensors rely on passive diffusion for analytes to reach receptors, but this group used directed particle motion toward sensor electrodes driven by AC dielectrophoresis [69]. An AC capacitive affinity sensor was developed in which low-voltage AC dielectrophoresis was used to carry analyte to the sensor via microfluidic movements that are independent of range and size of the analyte [69].

## 4. Piezoelectric Transduction

In comparison to optical and electrochemical transducers, piezoelectric transducers are a relatively recent addition to the repertoire of techniques for detecting small molecules, most popularly utilizing quartz crystal microbalances (QCM) [19]. Piezoelectricity, the phenomenon in which a material produces voltage under mechanical stress or vice versa, allows for the fabrication of sensors that utilize anisotropic crystals that oscillate upon the application of voltage [19,70]. Piezoelectric biosensors typically measure change in oscillation due to analyte binding for the measurement of analyte properties and kinetic information [70]. For example, in a conventional quartz crystal microbalance, the added mass upon binding increases the damping of the oscillation and a change in dissipation rate upon ceasing of voltage application that is related to the mass of the bound analyte following the Sauerbrey Equation [20,71]. Unfortunately, Piezoelectric transducers find difficulty in the measurement of small-molecule binding due to the mass dependency of the oscillation’s frequency change; additionally, though piezoelectric transducers are resistant to interference from non-transparent mediums compared to optical transducers, they are responsive to changes in viscosity [20]. Nevertheless, piezoelectric biosensors can be versatile and robust methods for small molecule detection and much progress has been made in enhancing the sensitivity of piezoelectric-based techniques. Furthermore, most relevant biosensing conditions require the sensor to be in liquid, which produces an additive damping to the measured frequency. Thus, the development of piezoelectric biosensors for small-molecule detection has been in amplifying the change of frequency upon binding or utilizing alternative means to collect data from the piezoelectric transducer.

**Figure 4:**
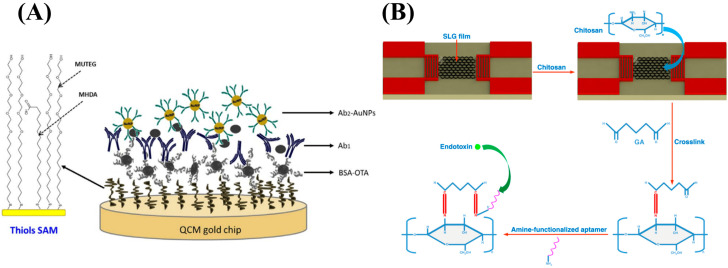
(**A**) Schematic of competitive immunoassay combined with QCM. Reprinted from [72], Copyright (2017), with permission from Elsevier. (**B**) Surface functionalization for shear horizontal SAW with graphene film for detection of endotoxin [76].

Quartz crystal microbalance is accepted as the most popular piezoelectric detection technique; however, though it displays great sensitivity, it struggles to have sufficient sensitivity for the detection of small molecules due to its signal dependency on mass and is prone to nonspecific binding. An indirect competitive strategy utilized gold nanoparticles conjugated with a secondary antibody that can then be bound to the captured primary antibodies on the sensor surface after competition between the antigen on the surface and the analyte as shown in Figure 4A [72]. The additional mass of the gold nanoparticle amplifies the change in frequency and dissipation that is produced from binding on the surface allowing increased ochratoxin detection sensitivity with a LOD of 4 × 10^−11^ g/mL in PBS and 1.6 × 10^−10^ g/mL in red wine [72]. This technique can be applied to other small-molecule analytes to enhance signal strength for QCM and other mass-dependent sensors like SPR. Another approach to compensate for the mass dependence of QCM is in the development of an electromagnetic piezoelectric acoustic sensor [73]. An ultrahigh frequency piezoelectric aptasensor was developed based on QCM that utilized excitation of an acoustic resonance with a piezoelectric quartz substrate through an external magnetic field induced by a spiral coil, which allows operation at high frequencies without metal contacts [73]. It is also one of the first uses of an aptamer immobilized on an organic adlayer for the detection of small molecules using an acoustic wave sensor, obtaining an LOD of 2.7 × 10^−7^ g/mL for cocaine [73]. The increased frequency decreases the penetration depth of the sensor allowing it to have higher sensitivity to binding events that occur at the biosensor surface [67]. Koutsoumpeli et al. utilized affimirs, an antibody mimetic, as a high-stability, low-cost alternative receptor surface on a self-assembled monolayer of long-chained alkanethiols with an oligoethylene glycol component [74]. The resulting monolayer provided resistance to nonspecific binding and was able to detect methylene blue with micromolar limit of detection despite not yet being optimized [74].

Another popular acoustic wave sensor is the surface acoustic wave (SAW) sensor, which utilizes similar technology, but the wave propagates only at the guiding layer of the substrate surface instead of the entire substrate allowing the use of higher frequencies and increasing sensitivity at the surface [75]. The most sensitive SAW sensors are Love-wave sensors that protect the interdigital transducer by the waveguide layer from harsh liquid environments [75]. Compared to QCM, thin film materials have not been widely explored, thus Sayago et al. investigated graphene oxide layers as an alternative to traditional gold film for the detection of chemical warfare agent (CWA) small molecules with LOD as low as 2 × 10^−7^ g/mL [8]. Similarly, a graphene oxide layer was produced with carbon vapor deposition, which is able to distinguish endotoxin from aflatoxin with aptamer receptors on a shear horizontal surface acoustic wave sensor as portrayed in Figure 4B, achieving a LOD of 2.53 × 10^−9^ g/mL [76].

Another piezoelectric-based transducer being explored is complementary metal oxide semiconductors that utilize a piezo-resistant membrane bridge sensor [77]. A microcantilever-based biosensor with a 2D array of suspended thin film and bridge structure offered higher sensitivity than traditional microcantilever biosensors, measuring phenytoin with an LOD of 4.06 × 10^−6^ g/mL [77]. Compared to SPR, the membrane bridge has increased reaction area and sensitivity, high stability due to increased stiffness, and its relatively small size makes it more compatible with point-of-care devices [77].

## 5. Conclusions

The label-free detection of small molecules has made great strides to meet the demands for diagnostics, drug development, and toxin detection with innovative optical, electrochemical, and piezoelectric techniques. This review covered some of the recent advancements and instrumentations developed to overcome the challenges of detecting low-mass small molecules in real-time. Optical techniques have developed methods to either enhance sensitivity until sufficiently detecting the low mass of small molecules or circumvent the issue through indirect measurement of binding or using methods dependent on charge rather than mass. Electrochemical techniques have expanded the repertoire of analytes that can be measured to small molecules that are not electroactive and/or have little to no charge while also fabricating surfaces that increase sensitivity and selectivity of conventional technologies. Piezoelectric techniques are exploring thin film surfaces that can be utilized to enhance their sensitivity and selectivity. A summary of all reviewed small molecule detecting biosensors is listed in Table 1.

Though these are great accomplishments advancing the field of label-free small-molecule detection, there remain challenges in implementing these technologies into widespread use. Optical techniques have limited options for surface chemistry beyond silane chemistry for glass surfaces and thiol chemistry for gold surfaces due to limitations for the surface’s opacity, roughness, and refractive index to preserve the optical properties necessary for sensor functionality. Electrochemical techniques face different complications as they are typically low-cost, portable, and easily implemented in assays or as lab-on-a-chip style sensors, but instead struggle with selectivity, particularly in complex environments such as serum. Impedimetric methods are also susceptible to high ionic concentrations, limiting their applications for biological studies. Piezoelectric techniques would benefit from improved surface chemistry that would allow them to avoid nonspecific binding in complex fluids such as serum and may be further optimized for sensitivity as its limitation to lower frequency waves is primarily due to substrate material.

## Figures and Tables

**Table 1:** Comparison of small-molecule detection biosensors.

Transducer	Technique	Analyte	LOD ^1^	Ref.
Optical	SPR	Estradiol	1.5 pg/mL	[31]
	SPR-based Oscillator	EMPPP	4.0 fg/mm ^2^	[33]
	GMR	DNP	75 ng/mL	[39]
	CAR	Furosemide	1.5 pg/mm ^2^	[34]
	CSOD	Imatinib	0.14 e^−^/µm ^2^	[41]
	Fluorescence	Picric acid	1.9 ng/mL	[44]
	Fluorescence	Uric acid	1.68 ng/mL	[46]
	Fluorescence	Glucose	8 mg/mL	[35]
	Liquid Crystal	L-histidine	7.8 mg/mL	[48]
Electrochemical	Amperometric	Biotin	0.9 µg/mL	[56]
	Voltammetry	Saxitoxin	0.28 ng/mL	[59]
	FET	Dehydroepiandosterone sulfate	16.5 ng/mL	[61]
	Quantum EIS	Cortisol	57.6 ng/mL	[65]
	EIS	Atrazine	0.22 zg/mL ^2^	[64]
	EIS	L-arginine	19.2 fg/mL	[66]
	RF Resonator	Glucose	0.11 ng/mL	[67]
	Capitative Affinity	Bisphenol A	2 fg/mL	[69]
Piezoelectric	QCM	Ochratoxin	0.4 pg/mL	[72]
	QCM-based	Cocaine	27 µg/mL	[73]
	SAW	Dipropylene glycol monomethyl ether	20 µg/mL	[8]
	SAW	Endotoxin	2.53 ng/mL	[76]
	MEMS-based	Phenytoin	4.06 µg/mL	[77]

^1^ Units converted to g/mL when possible. ^2^ Calculated for proposed sensor.
